# Elbow Fractures, Etc.

**Published:** 1881-10

**Authors:** Bernard Bartow


### Clinical Reports.

### Elbow Fractures.

### FRACTURE OF THE CONDYLES OF THE HUMERUS—TREATMENT BY THE STRAIGHT POSITION.

Reported by Bernard Bartow, M. D.

*Case 1.* May 14, 1881, A. B., æt. 4, fell from a height of four feet, striking upon her left elbow. I saw the patient six hours afterward and found the left elbow much swollen, the fore-arm flexed at an angle of 30°, the olecrannon projecting prominently, as in luxation, the lateral diameter of the joint increased, and movement of the fore-arm accompanied by pain. Further examination showed that the internal condyle was movable, crepitant and displaced. From the character of the crepitus it was evident that the fracture followed, in part, the line of the epiphysis. The attempt was made to crowd the condyle into its place, with the fore-arm extended; the fore-arm was then placed in the rectangular position, and the whole secured by a piece of belt leather moulded upon the back part and sides of the limb. After 48 hours the splint was removed to examine the joint. The swelling had subsided, but there was a recurrence of the displacement similar to that observed on my first visit. In addition, the muscles had contracted, fixing the parts as firmly as if it were purely a dislocation of the fore-arm.

The day following I examined the patient's elbow with the help of an anæsthetic, and then ascertained that both condyles were movable. Crepitus existed between them, as well as between the condyles and the end of the humerus. The diagnosis was, separation, and splitting, of the epiphysis. The spreading of the joint seemed to be due to the crowding apart of the condyles by the flexion of the fore-arm. Firm extension of the fore-arm permitted the easy replacement of the condyles—causing all deformity to disappear. While forced extension of the fore-arm was maintained by an assistant, a piece of moistened belt leather was moulded upon the limb, investing it completely with the exception of a space one inch wide upon its anterior surface. This was secured with a bandage. The adaptation of the leather to the contour of the limb was perfect; becoming firm in fifteen minutes, all tendency to displacement of the fractured parts was wholly overcome. The limb was maintained in the straight position for two weeks. Strong union having taken place at the end of that time, the fore-arm was flexed to the right-angle position and secured with the same splint, altered to a rectangular one, by cutting a V-shaped piece of leather from its sides opposite the elbow-joint. During the two following weeks the degree of flexion was gradually increased, until the extreme point to which the forearm could be bent, was reached. At the end of four weeks the splint was removed, permitting the limb to straighten; a bandage was the only support worn after that time. Six weeks from date of injury the functional recovery of the joint was nearly complete; a loss of about one-fifth of the flexion power was the only impairment of motion. (At the time of writing,—four months after the injury—the extent of motion in the elbow-joint of the affected limb, is, in all respects, as perfect as that of its fellow.)

*Case* 2. June 21, 1881, R. F., æt. 5, fell from a gate upon which he had been swinging, injuring his left elbow. The point upon which the blow was received could not be ascertained. He was attended by Dr. R. H. Hopkins shortly afterward, who diagnosed: fracture of the external condyle, and fracture of the internal epicondyle of the humerus. The line of fracture of the external condyle was partly epiphysial; the epicondyle separation was wholly epiphysial. The displacement of the external condyle was outward and backward; the head of the radius was dislocated and followed the condyle. There was marked increase of the lateral diameter of the joint. The fractured parts were adjusted, the fore-arm flexed at a right angle, and the whole secured with lateral splints of binder's board. Upon examination the second day, the appearance of the joint did not satisfy Dr. H., and he invited me to see the patient with him. The external condyle had become displaced, the head of the radius was prominent, the breadth of the joint was increased, and the fore-arm firmly fixed in its flexed position by the contraction of the muscles. I suggested dressing the fracture with the fore-arm in the straight position. After giving the patient ether, the fore-arm was forcibly extended. This allowed the condyle to be replaced, removing all deformity. While the fore-arm was extended, a leather splint was applied to the entire limb, as in *Case 1;* this secured the fracture perfectly against displacement. On the twelfth day, quite firm union having taken place, the fore-arm was flexed to a right-angle position, and the splint re-applied. The flexion was gradually increased, until the end of the fourth week, when the splint was removed, and a bandage substituted. The motions of the joint were wholly restored at the end of the sixth week. There was no displacement at the points of fracture. In both instances the "carrying function" of the limb was unimpaired.

In a recent paper* Dr. Allis, of Philadelphia, has shown, in a very able manner, why deformity and anchylosis follow the employment of rectangular splints in the treatment of fractures through the condyles of the humerus. He recommends that such fractures be treated with the forearm in the extended position, with a view to preventing displacement of the condyles. In the foregoing cases the treatment has been essentially in accordance with his views. The excellent results obtained in both instances is a good illustration of the correctness of his views.

In the matter of an appliance I prefer a leather splint to the "egg-paste" bandage, or adhesive strips, used by Dr. Allis. The leather can be moulded to fit the arm as perfectly as a paste bandage, requires less time and inconvenience, is stronger, and can be sprung off the arm with great ease for examination of the fracture.

---

*Trans. Anat. Soc., Jan., 1880.

In fracture through the condyles, firm union usually takes place in two weeks. It is unnecessary, therefore, to maintain the limb in the straight position beyond that time. (Dr. Allis recommends that the straight position be maintained for thirty days.) Flexion of the fore-arm at the end of two weeks causes no displacement of the condyles; and by loosening the tissues from adhesions, places the joint in a better condition to resume functional activity, when all restraint shall have been removed.

### SEPARATION OF THE EPIPHYSIS OF THE LOWER END OF THE HUMERUS.

M. H., aged 6, fell from a velocipede, striking upon the left elbow. She was attended at once by a homœopathic surgeon, who obscurely diagnosed an "elbow fracture" and dressed it with an anterior rectangular splint. The patient was seen by Dr. H. R. Hopkins and myself, eighteen hours after the accident. Then the appearance of the elbow closely resembled luxation of the bones of fore-arm, backward. The muscles had contracted, rendering the fore-arm almost immovable. The patient was etherized, and it was ascertained that there was complete separation of the lower epiphysis of the humerus. The fragment followed the movements of the fore-arm. Crepitus could not easily be obtained by flexion or extension of the fore-arm; but, with the fore-arm flexed, it could be produced easily, by grasping the condyles and moving the fragment from side to side. The tendency of the posterior projection to recur, when flexion was relaxed, and the complete disappearance of the signs of dislocation, when the fore-arm was extended and flexed, confirmed the diagnosis. The fore-arm was placed, semi-prone, in the right-angle position, and the limb dressed in a moulded leather splint embracing its posterior and lateral surfaces. After three days the angle was changed to that of extreme flexion, which angle was maintained until the eighth day, when the fore-arm was extended at an angle of 40°. On the fourteenth day union was found to be quite firm, and the fore-arm was brought down to a position of full extension. Passive motion has been practiced up to the present time (thirty days from date of injury). There is no displacement of the epiphysis; flexion and extension are nearly perfect—the only obstacle to free motion being an abundance of inflammatory exudates.

The case is interesting when compared with *Case 1*, in which the epiphysis was splint, necessitating a radically different mode of treatment.

